# Prognostic significance of YKL-40 expression in canine cutaneous mast cell tumors

**DOI:** 10.1186/s12917-024-04385-1

**Published:** 2024-11-29

**Authors:** Chien-Chun Kuo, Wei-Hsiang Huang, Su-Ya Yang, Yen-Chen Chang, Hui-Wen Chang, Chian-Ren Jeng, Jih-Jong Lee, Albert Taiching Liao

**Affiliations:** 1 Department of Veterinary Medicine, School of Veterinary Medicine, National Taiwan University, No. 1, Sec. 4, Roosevelt Road, Taipei, 106319 Taiwan (ROC); 2 Animal Cancer Treatment Center, National Taiwan University Veterinary Hospital, National Taiwan University, No. 153, Sec. 3, Keelung Road, Taipei, 106328 Taiwan (ROC); 3 Graduate Institute of Molecular and Comparative Pathobiology, School of Veterinary Medicine, National Taiwan University, No. 1, Sec. 4, Roosevelt Road, Taipei, 106319 Taiwan (ROC); 4 Institute of Veterinary Clinical Sciences, School of Veterinary Medicine, National Taiwan University, No. 1, Sec. 4, Roosevelt Road, Taipei, 106319 Taiwan (ROC)

**Keywords:** YKL-40, Canine cutaneous mast cell tumor, Histological grade, Immunohistochemistry, Prognosis

## Abstract

**Background:**

YKL-40, a secretory glycoprotein, is involved in tumor cell proliferation, metastasis, and angiogenesis in human cancers. Its overexpression has been correlated with unfavorable prognosis in many human cancers. In veterinary medicine, elevated YKL-40 levels in the serum of canine cutaneous mast cell tumors (cMCTs) were observed in our previous study. However, the expression pattern of YKL-40 in canine cMCT tissues, along with its association with clinical and pathological features, is still unknown. This study aims to retrospectively investigate the expression level of YKL-40 in the tissues of canine cMCTs and its correlation with clinical features, pathological characteristics, and clinical outcomes. Forty formalin-fixed paraffin-embedded cMCT tissues collected from forty dogs were diagnosed as low-grade (*n* = 20) or high-grade s(*n* = 20) MCT according to the Kiupel grading system. The expression level of YKL-40 in cMCT tissues was investigated using immunohistochemical staining and immunoreactivity score (IRS).

**Results:**

YKL-40 was expressed in all cMCTs at different levels, with significantly stronger expression in low-grade cMCTs compared to high-grade cMCTs. The expression level was also associated with tumor diameter, histological grade, mitotic counts, vessel density, and survival of cMCTs. The overall survival of cMCT dogs showed significant differences (*p* < 0.01) among mild (*n* = 15, MST 219 days), moderate (*n* = 19, MST not reached), and high (*n* = 6, MST not reached) YKL-40 expression groups. Among low-grade cMCTs, overall survival was significantly different between mild YKL-40 expression (MST 319 days) and moderate to high YKL-40 (MST not reached) expression (*p* < 0.01). In high-grade cMCTs, overall survival was not correlated with YKL-40 expression (*p* = 0.6589).

**Conclusions:**

This study found that the YKL-40 expression level was significantly stronger in low-grade than in high-grade canine cutaneous mast cell tumors and was associated with various clinical and pathological features. Stronger YKL-40 expression level correlated with longer survival time, especially in low-grade cMCTs. Therefore, YKL-40 could serve as a prognostic marker for cMCTs.

**Supplementary Information:**

The online version contains supplementary material available at 10.1186/s12917-024-04385-1.

## Background

Cutaneous mast cell tumor (cMCT) is a common neoplasm in dogs. The biological behavior of cMCT is variable and could be related to the differentiation of neoplastic cells. Several prognostic features of cMCT have been reported, including clinical stage [1], histological grade [1–3], mitotic index [1, 3], KIT expression pattern, and *c-kit* gene mutation status [1, 4]. Proliferation and inflammatory markers, including PCNA [5], Ki67 [5–7], and COX-2 [7], were also applied to predict prognosis [4, 8] of canine cMCT. Although none of them could precisely predict the survival time, 2-tier [3] and 3-tier [2] histopathologic grading systems were the most common histopathological factors to predict the progression of canine MCTs.

YKL-40 is a chitinase-like protein that plays a role in cancer proliferation [9, 10], metastasis [11–14], and angiogenesis [15–19]. This glycoprotein could be secreted by epithelial cells, inflammatory cells [20], and neoplastic cells [21]. A variety of human tumors can express and secrete YKL-40 [21], including squamous cell carcinoma [22], melanoma, breast cancer [23], and non-Hodgkin lymphoma [21]. Recently, the expression level in neoplastic cells and circulation has been studied as a prognostic marker in human cancers [19]. In human bladder cancer [24, 25], gastric cancer [26], and glioblastoma [27], the expression of YKL-40 in neoplastic cells has been correlated with a higher histological grade and poor prognosis. In urothelial carcinoma, higher expression of YKL-40 is correlated with advanced features and a shorter survival time [25]. Elevation of serum YKL-40 in patients with renal cell cancer was correlated with a shorter survival time [28]. Besides, YKL-40 expressed by tumor-infiltrating immune cells may also be related to tumor survival rates. For example, reduced survival in human colorectal cancer is associated with YKL-40 expression in immune cells but not with colorectal tumor cells [29]. In addition, the expression levels of YKL-40 in the blood and neoplastic cells may have different meanings. For instance, in ovarian cancer, serum YKL-40 is associated with survival time, but YKL-40 expression in neoplastic cells is not related to the survival time [30]. Overall, the YKL-40 protein is considered a potential prognostic biomarker for certain types of human cancer.

On the other hand, YKL-40 has been found in the cytoplasm of human mast cells [20, 31]. It may be involved in fibrosis of smooth muscle in the urinary bladder [31] and extracellular matrix remodeling in asthma [32]. In veterinary science, higher blood concentrations of YKL-40 have been found in the serum of dogs with MCT [33]. However, there is a lack of research focusing on the YKL-40 expression level and neoplastic features in canine cMCTs. Therefore, this study aims to investigate the association of YKL-40 expression levels in canine cMCT tissues with their clinical and histopathological features.

## Results

### Identification of antibody specificity

**Fig. 1:**
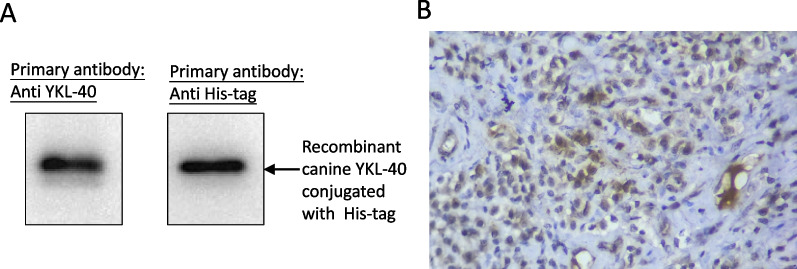
Canine YKL-40 can be detected by the anti-human YKL-40 antibody used in this study. **A** The canine recombinant YKL-40 (MW: 46 kDa) was separated on 10% polyacrylamide gel and transferred to a PVDF membrane for immunoblot analysis. Rabbit anti-human YKL-40 antibody (1:5000, Cat. A3166, ABclonal, USA; left blot) and mouse anti-his antibody (1:5000, Abcam, USA; Right bolt) were respectively applied to access their immunoreactivity on canine recombinant YKL-40. The cropped blots are presented in this figure, and the full-length blots are presented in Supplementary Fig. 1. **B** Immunohistochemical staining with hematoxylin counterstaining was performed on the tissue of a dog with dermatitis with mastocytosis. Immunoreactivity (brown color; rabbit anti-human YKL-40 antibody, 100x) of YKL-40 was present at moderate intensity in the cytoplasm of mast cells

At the beginning of this study, the specificity of the rabbit anti-human YKL-40 antibody against canine YKL-40 was investigated. Figure 1A shows that both anti-human YKL-40 antibody and anti-His tag antibody can be used to probe canine recombinant YKL40 in Western blot. The tissue section of canine dermatitis with mastocytosis was stained by IHC using this antibody. Positive immunoreactivity was found in the cytoplasm of mast cells with moderate intensity (intensity = 2) observed in non-neoplastic mast cells (Fig. 1B).

### Characteristics of the included cases

Forty canine cMCTs (20 low- and high-grade tumors) were included in this study. These samples were collected from 14 male dogs (4 intact and 10 neutered) and 26 female dogs (1 intact and 25 neutered). The median age was 11 years (range: 5–15 years). The dog breeds included 16 mixed-breed dogs, 5 Labrador retrievers, 4 Beagles and 4 Schnauzers, 2 Golden retrievers, 2 Poodles, 2 Shi Tzus, 1 Bull Terrier, 1 Chihuahua, 1 Sheltie, 1 Shiba and 1 Yorkshire Terrier. Five high-grade MCT cases received medical therapy before surgical excision, with medication stopped at least one week before the surgical excision. One patient received glucocorticoid therapy, and the remaining four patients received a combination of glucocorticoid and chemotherapy.

The median diameter of cMCTs was 5 cm (range: 0.6–18 cm). The median tumor diameter was larger in high-grade cMCT (7.0 cm; range: 1.2–18 cm) than in low-grade cMCT (1.9 cm; range: 0.6–7.0 cm) (*p* < 0.01). The anatomic locations of the tumors included the limbs (*n* = 16), trunk (*n* = 23) and head (*n* = 1). Thirty-three dogs had a single tumor, and 7 dogs had multiple MCTs that had been excised at the same time. Tumor ulceration was recorded in 15% (*n* = 6) of cases. Regional lymph node metastasis was recorded in 30% (*n* = 12) of cases examined by fine needle aspiration or surgical pathology. Distant metastasis was recorded in 5% (*n* = 2) of cases.

These patients were categorized into WHO clinical stages, including 22 stage I, 8 stage II, 8 stage III, and 2 stage IV. When using 5 mitotic counts (MC) per 10 high power fields as the cutoff value, 24 cases had fewer than 5 MC, and 16 cases had more than 5 MC. The median mitotic counts were higher in the high-grade group than in the low-grade groups (*p* < 0.01). The median vessel density was also higher in the high-grade MCT group than in the low-grade MCT group (*p* = 0.04). The breed, sex, age, number of masses, anatomic location, tumor ulceration, and WHO clinical stage were not significantly different between low- and high-grade MCTs. Detailed comparisons between the low and high-grade groups are shown in Table 1.

**Table 1:** Clinical and histological features of low-grade and high-grade canine cMCTs

**Histological grade**	**Low-grade (*n* = 20)**	**High-grade (*n* = 20)**	*P*
Age, years			
Median (IQR)	10 (8–12)	12 (10–14)	0.1106
Breed			
Mixed	7	9	0.7475
Pure	13	11	
Sex			
Male	7	7	> 0.9999
Female	13	13	
Numbers of Mass			
Single	18	15	0.4075
Multiple	2	5	
Anatomic location			
Limbs	10	6	0.2136
Trunk	9	14	
Head	1	0	
Tumor diameter, cm			
Median (IQR)	1.9 (1.0–5.4)	7.0 (4.6–11.5)	< 0.01
Ulceration			
No	19	15	0.1818
Yes	1	5	
Lymph node involvement			
No	17	11	0.0824
Yes	3	9	
WHO Clinical stage			
I	15	7	0.0599
II	3	5	
III	2	6	
IV	0	2	
Mitotic counts/10 HPF			
Median (IQR)	1 (1–2)	10 (5–12)	< 0.01
MC ≤ 5	19	5	< 0.01
MC > 5	1	15	
Vessel density/10 HPF			
Median (IQR)	22 (16–38)	37 (23–44)	0.0377
YKL-40 expression level, IRS			
Median (IQR)	6 (6–9)	3 (3–6)	< 0.01
YKL-40 expression level			
Mild	3	12	< 0.01
Moderate	11	8	
Strong	6	0	

*IQR* Interquartile range, *HPF* High power field (400x), *MC* Mitotic count, *IRS* Immunoreactivity score

### YKL-40 expression levels in low- and high-grade canine cMCTs

**Fig. 2:**
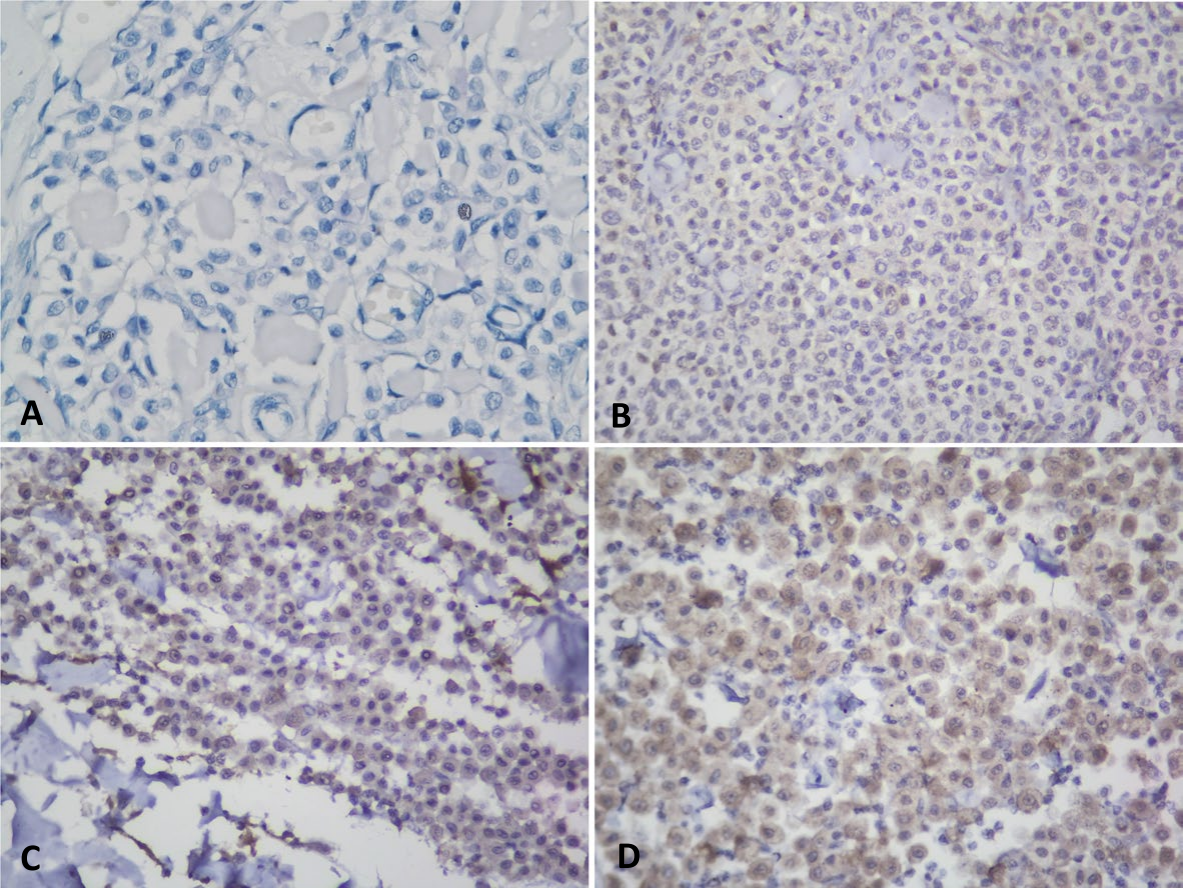
The intensities of YKL-40 immunoreactivity in canine cMCTs. The cytoplasm of YKL-40 positive mast cells is labeled brown in DAB staining. Different colorations represent different staining intensities. **A** Negative staining intensity of negative control of a low-grade MCT (Case No.19). **B** Mild staining intensity of YKL-40 was detected in the cytoplasm of a high-grade MCT (Case No.2) (**C**) Moderate staining intensity of YKL-40 was detected in the cytoplasm of a low-grade MCT (Case No. 16). **D** Intense staining intensity of YKL-40 was detected in the cytoplasm of a low-grade MCT (Case No.19)

YKL-40 was detected in the tumor cells of all canine cMCT tissues using IHC staining. The staining intensity and proportion varied among different cases. The staining intensity was based on the coloration of cytoplasmic signal and could be scored from 0 (negative), 1 (mild), 2 (moderate) to 3 (intense). Representative staining intensities are shown in Fig. 2. The median IRS of YKL-40 expression in all canine cMCTs was 6 (range: 2—10).

**Fig. 3:**
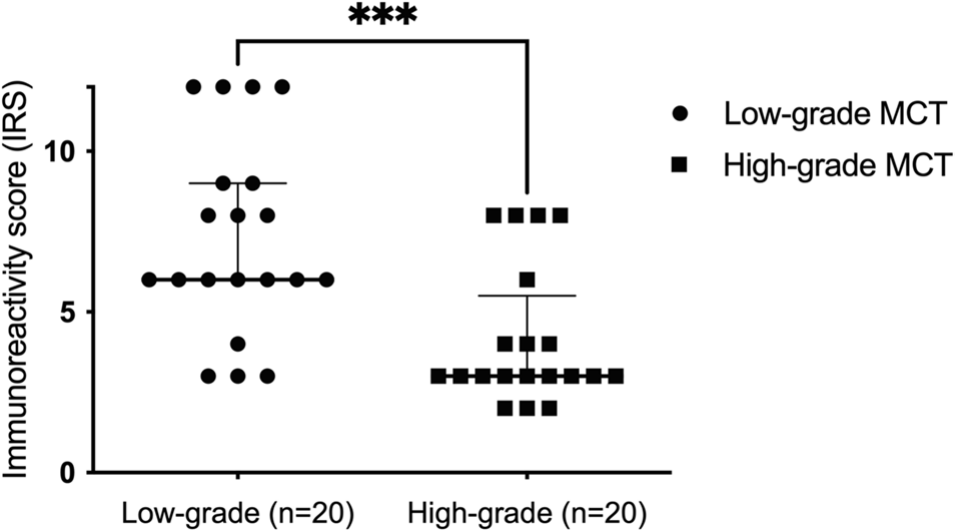
The immunoreactive score (IRS) of YKL-40 expression in low- and high-grade canine cMCT tissues. Histological low-grade cMCTs expressed mild to high levels of YKL-40 (median IRS = 6). High-grade cMCTs expressed mild to moderate levels of YKL-40 (median IRS = 3). The expression level of YKL-40 was significantly stronger in low-grade cMCTs than in high-grade cMCTs (*P* < 0.01)

In low-grade cMCTs, mild (15%, *n* = 3), moderate (55%, *n* = 11), and strong (30%, *n* = 6) YKL-40 expression levels were observed. The median IRS of YKL-40 expression in low-grade cMCTs was 6 (range: 3–12). In high-grade cMCTs, the IRS of the YKL-40 expression ranged from mild (60%, *n* = 12) to moderate (40%, *n* = 8). The median IRS of YKL-40 expression in high-grade cMCTs was 3 (range: 2–8). Low-grade cMCTs expressed stronger YKL-40 than high-grade cMCTs (*p* < 0.01). The scatter plot of IRS of low- and high-grade cMCTs is shown in Fig. 3.

There was no significant difference in IRS between patients who received treatment before surgery (*n* = 5, median IRS = 3) and those who did not receive any treatment before surgery (*n* = 15, median IRS = 3) in high-grade cMCTs (*p* = 0.9518).

### The correlation between YKL-40 expression levels and prognostic indicators of canine cMCTs

To understand the correlation of YKL-40 expression levels with various prognostic indicators of canine cMCTs, these cases were categorized into two groups: mild YKL-40 expression and moderate/strong YKL-40 expression. The mild YKL-40 expression group comprised 12 high-grade and 3 low-grade MCTs, while the moderate/strong YKL-40 expression group was composed of 17 low-grade and 8 high-grade MCTs (Table 1). The moderate/strong expression group had a higher proportion (17/25) of low-grade cMCTs compared to the mild expression (3/15) group (*p* < 0.01).

The median tumor diameters were larger in the mild YKL-40 expression group (median = 7.0 cm, range: 2.5–12.0 cm) than in the moderate/strong expression group (median = 4.2 cm, range: 1.0–18.0 cm) (*p* = 0.0138). The median mitotic counts were higher in the mild YKL-40 expression group (median = 8, range: 1–40) compared to the moderate/strong expression groups (median = 1, range: 0 -19) (*p* = 0.03). The median vessel density was higher in the mild YKL-40 expression group (median = 38, range: 19–40) compared to the moderate/strong YKL-40 expression group (median = 21, range: 12–47) (*p* = 0.013). Detailed comparisons between the mild and moderate/strong YKL-40 expression groups are shown in Table 2.

**Table 2:** Association of YKL-40 expression levels between clinical and pathological parameters

YKL-40 Expression	Mild (*n* = 15)	Moderate/ Strong (*n* = 25)	*P*
Age, years			0.6569
Median, IQR	12 (8.5–13.25)	11 (8–13)	
Sex			
Male	6	8	0.7356
Female	9	17	
Tumor location			
Limbs	10	13	0.5166
Trunk	5	11	
Head	0	1	
Tumor diameter, cm			
Median (IQR)	7.0 (2.5–12.0)	4.2 (1.0–18.0)	0.0138
Ulceration			
No	13	20	0.4983
Yes	2	4	
Lymph node involvement			
No	9	18	0.7377
Yes	6	7	
WHO Clinical stage			
I	8	14	0.9831
II	3	5	
III	4	6	
IV	1	1	
Histological grade (2-tier)			
Low	3	17	< 0.01
High	12	8	
Mitotic counts/10 HPF			
Median (IQR)	8 (3–10)	1 (1–19)	0.0294
MC ≤ 5	6	18	0.0939
MC > 5	9	7	
Vessel density/10 HPF			
Median (IQR)	38 (26–49)	21 (16–39)	< 0.01

*IQR* Interquartile range, *HPF* High power field (400x), *MC* mitotic count

### The correlation between survival time and tumor grading of canine cMCTs

In the high-grade group, fourteen patients received adjuvant therapy, including chemotherapeutic medication and/or target therapy, while the other six patients received palliative treatment. Two high-grade cMCT cases with low clinical stages (I and II) were alive at the end of the study. Both cases received wide-surgical excision followed by chemotherapy. The survival time of high-grade cMCT cases ranged from 18 to 1060 days. The median survival time (MST) of high-grade patients was 180 days. The overall survival for high-grade cases with chemotherapy (MST = 176 days; n = 14) was longer than for high-grade cases without chemotherapy (MST = 126 days; n = 6), but the difference was not significant (*p* = 0.4597).

In the low-grade group, eighteen patients did not receive chemotherapeutic medication after surgery. Two patients received chemotherapeutic medication post-surgery because of regional lymph node metastasis identified in the pathological report. Three patients died of cMCT progression; one of these received chemotherapeutic medication, while the others did not. Ten patients were alive at the end of the study, and seven patients died of other diseases. The survival time of low-grade cMCT cases ranged from 235 to 1264 days; the median survival time could not be estimated because 85% (*n* = 17) of cases were censored. The overall survival between low-grade cases with treatment (*n* = 2, MST = 837 days) and low-grade cases without treatment (*n* = 18, MST not reached) was not significantly different (*p* = 0.2071).

**Fig. 4:**
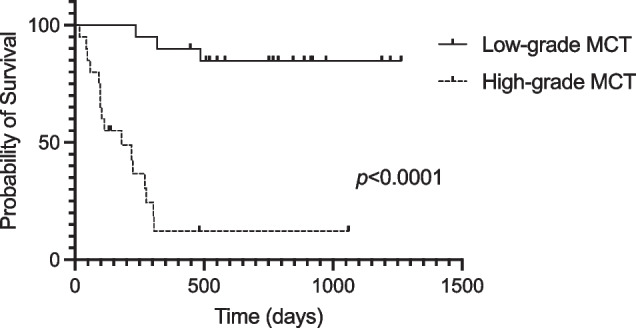
Kaplan‒Meier overall survival curves comparing dogs with low- and high-grade cMCTs. The sold line represents low-grade MCTs (*n* = 20), and the dashed line represents high-grade MCTs (MST = 180 days, *n* = 20). Vertical lines denote dogs that were censored from survival analysis

The median follow-up time was 383 days (range: 18–1264 days). The overall survival between high-grade cases and low-grade cases was significantly different (*p* < 0.0001) in the log-rank test. The Kaplan‒Meier survival curve comparing dogs with low- and high-grade cMCT is shown in Fig. 4.

### The correlation between survival time and YKL-40 expression levels of canine cMCTs

When cases were grouped based on their YKL-40 expression level, mild expression cMCTs comprised 15 cases, including 12 high-grade and 3 low-grade MCTs. Moderate expression cMCTs included 8 high-grade and 11 low-grade MCTs. Strongly expressed cMCTs consisted only of 6 low-grade MCTs. The median survival time was 219 days for dogs with mild YKL-40 expression. The median survival time could not be estimated for dogs with moderate and strong YKL-40 expression because the probability of survival exceeded 50% in dogs with moderate YKL-40 expression, and all the cases were censored in dogs with strong YKL-40 expression.

**Fig. 5:**
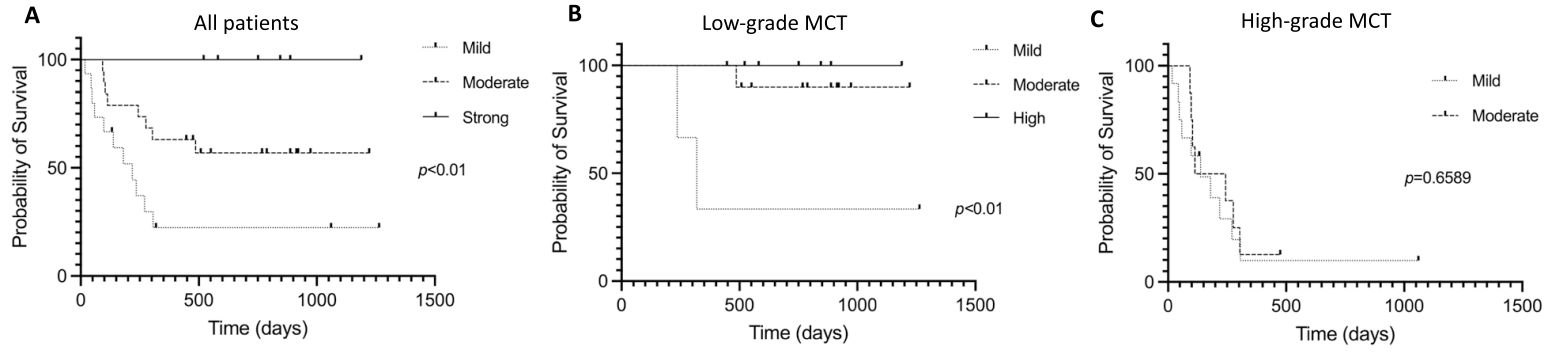
Kaplan‒Meier overall survival curve comparing different YKL-40 expression levels between all patients (5**A**), dogs with low-grade MCT (5**B**), and dogs with high-grade MCT (5**C**). The sold line, dashed line, and the dotted line represent strong, moderate, and mild YKL-40 expression level of MCTs. Vertical lines denote dogs that were censored from survival analysis of these curves

The overall survival was significantly different (*p* < 0.01) between mild, moderate, and strong YKL-40 expression in the log-rank test. The Kaplan‒Meier survival curve comparing the YKL-40 expression level in cMCT dogs is shown in Fig. 5A.

### The correlation among survival time, tumor grading, and YKL-40 expression levels of canine cMCTs

Among low-grade cMCTs, the MST of dogs with mild YKL-40 expression was 319 days. The median survival time could not be estimated for dogs with moderate and strong YKL-40 expression because the probability of survival exceeded 50% in dogs with moderate YKL-40 expression, and all the cases were censored in dogs with strong YKL-40 expression. The overall survival among the low-grade cMCT cases with mild, moderate, and high YKL-40 expression was significantly different in the log-rank test (*p* < 0.01). The Kaplan‒Meier survival curve comparing dogs with low-grade cMCTs with different YKL-40 expression levels is shown in Fig. 5B.

Among high-grade cMCTs, the MST of dogs with mild and moderate YKL-40 expression was 138 days and 179 days, respectively. Overall survival was not significantly different in the log-rank test. The Kaplan‒Meier survival curve comparing the dogs with high-grade MCTs with different YKL-40 expression levels is shown in Fig. 5C.

## Discussion

YKL-40 naturally exists in the cytoplasm or cytoplasmic granules of human mast cells [31] and could be involved in the inflammatory function of active mast cells [31, 32]. In this study, moderate YKL-40 immunoreactivity was found in the cytoplasm of mast cells in the tissues of canine dermatitis with mastocytosis (Fig. 1), indicating that YKL-40 can be produced by canine mast cells and may participate in the inflammatory function of active mast cells. The intensity of YKL-40 in mast cells was similar to that observed in human study [20]. Additionally, we found that canine cMCTs could produce variable amounts of YKL-40 (Fig. 2). To our knowledge, this is the first study to investigate the expression level of YKL-40 in canine mast cells and cMCTs.

In this study, the included low- and high-grade cMCT cases share similar clinical and pathological features to other studies [1, 3, 7]. These high-grade cMCT cases had a poorer prognosis, more mitotic counts, larger tumor size, and higher vessel density compared to the low-grade cMCT cases (Table 1). Therefore, the grouping method based on the 2-tier histological grade appears to be a suitable design for analyzing the relation of prognostic markers with canine cMCT and could minimize the prognostic effect of treatment. Based on this grouping, the study also investigated the correlations between YKL-40 expression levels and other indicators, including clinical features, pathological characteristics, and survival.

We found that the expression levels of YKL-40 varied with different histological grades of cMCTs. Compared to previous studies, it has been observed that YKL-40 expression levels could differ among various histological subtypes of cancers, including thyroid, colorectal, gastric, and ovarian cancer [21]. For example, differences in expression levels were noted between non-neoplastic cells and neoplastic cells in mammary gland carcinoma [34]. In addition, expression levels in the breast in situ carcinomas and invasive carcinomas are higher than in normal breast epithelial cells [23]. These studies have found increased YKL-40 expression in malignant tumor cells.

However, our study found that high-grade cMCTs tend to express milder levels of YKL-40, while low-grade cMCTs express stronger levels of YKL-40 (Fig. 3). This result contrasts with findings from other tumor studies [21, 23, 34] and our hypothesis, which assumed that more malignant mast cell tumors would express stronger levels of YKL-40. Since this is the first study investigating YKL-40 in mast cell tumors and lacks related human studies, we speculate that this result may be related to the nature of mast cells. Mast cells could express moderate amounts of YKL-40, which might be involved in the inflammatory response [31, 32]. Therefore, when mast cell tumors become poorly differentiated, their YKL-40 expression decreases. As a result, high-grade MCTs, which are regarded as poorly differentiated MCTs, exhibit milder YKL-40 expression levels compared to low-grade MCTs.

This study found that preoperative treatment, followed by a one-week discontinuation period before surgery, does not affect tissue YKL-40 expression. We compared the YKL-40 expression levels in high-grade MCT dogs who received preoperative treatment to those who did not receive treatment and found no significant difference in YKL-40 expression in mast cell tumor tissues between these two groups. Although studies have shown that steroid use may affect circulating blood levels of YKL-40, it is also related to the severity of the disease [35]. In this study, the washout period may also ensure that this did not influence the analysis of YKL-40 expression levels. Additionally, research indicating that steroids do not impact mast cell tumor grading has been reported [36]. Since all high-grade MCT patients in our study who received preoperative steroids had a discontinuation period before surgery, preoperative steroid use with a subsequent washout period does not affect the histological grading of the patients in this study.

In the present study, a stronger YKL-40 expression level in cMCT was associated with a favorable prognosis (Fig. 5A), indicating that YKL-40 could be a prognostic marker for canine cMCTs. Interestingly, mild YKL-40 expression was correlated with a poor prognosis in low-grade cMCTs but not for high-grade cMCTs (Fig. 5B and C). We suggest that other more significant factors may influence the tumor progression and prognosis of high-grade MCT. Low-grade and high-grade cMCT patients had similar clinical features, including clinical stage, and received wide-margin surgical excision without chemotherapeutic treatment. These findings support that the prognosis of cMCTs was related to the differentiation of neoplastic cells. Therefore, the YKL-40 expression level, which is related to the differentiation of neoplastic cells, could be proposed as a valuable prognostic marker to predict the outcome, especially in low-grade canine cMCTs.

The histological features were also related to the YKL-40 expression level (Table 2). Stronger YKL-40 expression was associated with lower mitotic counts and smaller tumor size, indicating that YKL-40 may not be a proliferative biomarker for canine cMCTs. Interestingly, the higher YKL-40 expression was related to lower vessel density, which indicated that YKL-40 might not be involved in angiogenesis in cMCTs. Vessel density has been reported as a prognostic marker [37], but not one of the criteria for the histological grade of MCT. High-grade cMCTs present more vessel density and could be associated with larger tumor size and poorer prognosis. Previously, YKL-40 had the ability to promote angiogenesis in various human cancers [15–19]; therefore, we believe that the YKL-40 level could be positively correlated with the vessel density of canine cMCT. Although the results conflict with our hypothesis, we suggest that YKL-40 could be involved in the normal biological function of mast cells but not the proliferation of MCT cells or angiogenesis in MCT tissues.

The expression level of YKL-40 was semi-quantified by immunohistochemical staining which may have been affected by the study technique. We confirmed the cross-species reactivity of the anti-human YKL-40 antibody against canine YKL-40 by western blot and IHC staining. Nonspecific binding was not detected at the proper antibody concentration. The IHC protocol was also standardized to evaluate the staining intensity. We used whole tissue slide instead of tissue microarray [21] to evaluate different regions of mast cell tumors, including the central area, surgical margin, and dermal area. Heterogeneity of expression level was commonly found; therefore, we observed the hotspot area to represent the YKL-40 expression level of the tumor. The expression level was semi-quantified by the immunoreactivity score (IRS) system [38], which was applied in the YKL-40 expression study [22]. This method is suitable for evaluating heterogeneous tumors [7, 39].

There are several limitations to this study. The retrospective nature of the study led to variability in medical records and treatment protocols. Censored data was noted in most of the dogs with low-grade MCT due to their survival at the end of the study, which may influence the overall survival. Second, the sample size was small and may not reflect the epidemiology of MCTs. Third, although an equal number of low- and high-grade patients was compared knowing histological prognostic factors, this could not represent the actual percentage of low- and high-grade mast cell tumors in our database. Although the FFPE tissue was fixed as soon as possible and preserved under the same conditions, protein degradation may be a concerning factor in these samples [40].

## Conclusion

Canine cMCTs can generally produce varying amounts of YKL-40. The YKL-40 expression level is significantly lower in canine cMCTs that have larger tumor diameters, higher mitotic counts, higher vessel density, and histological high-grade tumors. A moderate to strong YKL-40 expression level in canine cMCTs is usually associated with a good prognosis in patients. In histological low-grade canine cMCTs, a mild YKL-40 expression level is correlated with a poor prognosis, indicating that the YKL-40 expression level can be a valuable prognostic marker for canine cMCTs, especially in low-grade canine cMCTs.

## Methods

### Case selection

The medical and pathological records of dogs who underwent excised primary MCTs from 2017 to 2021 were retrospectively searched at the National Taiwan University Veterinary Hospital (NTUVH), Taipei, Taiwan. After surgical excision, the tissues were immediately fixed in 10% neutral buffered formalin and embedded in paraffin. All samples were analyzed for pathological diagnosis by at least two veterinary pathologists at the Graduate Institute of Molecular and Comparative Pathobiology (GIMCP) of NTU. Eighty-one MCTs were found in the GIMCP and NTUVH database, and non-cutaneous MCTs and recurrent MCTs were excluded. Twenty high and twenty low-grade cMCTs, which were diagnosed according to the Kiupel grading system [3], were randomly selected and involved in this study.

Patient information, including breed, age, sex, neutered status, tumor location, number of tumors, tumor diameter, ulceration, cytological or histological examination of regional lymph nodes, abdominal ultrasound, thoracic radiography, and WHO clinical stage [41], were collected from the medical records. The largest or higher grade MCT was represented as a major lesion for further analysis when multiple MCTs were recorded in the same patient. Pathological information, including mitotic counts per 10 high power fields (400x) and lymph node involvement, were also collected from the pathological reports. The study was reviewed and approved by the ethics committee of the Institutional Animal Care and Use Committee of National Taiwan University (Permit number: NTU-109-EL-00169). All the owners signed the informed consent forms.

### Western blot

To confirm the species cross-reactivity of commercial anti-human YKL-40 polyclonal antibodies to canine YKL-40, western blotting was performed using recombinant canine YKL-40 protein [33]. A primary antibody, rabbit polyclonal anti-human YKL-40 antibody (1:1000, Cat. A3166, ABclonal Inc, USA) was used to detect canine YKL-40. The secondary antibody was goat anti-rabbit IgG (H + L) (1:5000, Cat. E-AB-1003, Elabscience, Texas, USA). The recombinant canine YKL-40 protein (rcYKL-40) was separated in 10% sodium dodecyl sulfate‒polyacrylamide gel electrophoresis gels (SDS‒PAGE). The protein was transferred to a polyvinylidene difluoride (PVDF) membrane and blocked in Tris-buffered saline containing 5% non-fat milk at room temperature for 1 h. The membrane was incubated with primary antibody (1: 1000) at 4 °C overnight. The membrane was subsequently incubated with secondary antibodies (1:5000) at room temperature for 1 h. The target proteins were detected using Immobilon Western Chemiluminescent HRP Substrate (Merck, Darmstadt, Germany) and photographed using a MultiGel 21 gel image system (TOP BIO Co., New Taipei City, Taiwan).

### Immunohistochemical (IHC) staining

Four micrometers thick formalin-fixed, paraffin-embedded tissues were prepared for the immunohistochemical analysis. Following the dewaxing and dehydration of the tissue sections, the antigen retrieval was performed at 90 °C for 30 min with an EDTA-based retrieval solution (Trilogy, Cat. 920P-06, Millipore Sigma, United States). A commercial anti-mouse/anti-rabbit detection kit (Cat. RE7260-CE, Novolink Polymer Detection System, Leica Biosystems, United States) was used for blocking endogenous peroxidase, blocking non-specific binding, and secondary antibodies according to the manufacturer’s instructions. The tissue slide was incubated with appropriate primary antibodies for 1 h in a humid chamber at room temperature. The primary antibodies were polyclonal rabbit anti-YKL-40 (1:100, Cat. A3166, ABclonal Inc, United States) and monoclonal mouse anti-human CD31 (1:100, Cat. M0823, Agilent, United States). Immunoreactivity was visualized with the 3,3'-diaminobenzidine chromogen and DAB Substrate buffer (Novolink Polymer Detection System, Leica Biosystems, United States). The other cellular features were contrasted with hematoxylin for 1 min. For negative controls, the primary antibody was replaced by phosphate-buffered saline. The blood vessel was identified by the vessel lining cells, which presented as the immunoreactivity against CD31.

### YKL-40 expression analysis

The expression level of YKL-40 was semi-quantified by the immunoreactivity score (IRS) [39], which was calculated by multiplying the percentage of positive cells by the staining intensity. The percentage of immunoreactivity was counted within 100 tumor cells and then scored as 0 (0%), 1 (less than 20%), 2 (20% to 50%), 3 (50% to 80%), and 4 (greater than 80%). The staining intensity was scored as 0 (no reaction), 1 (weak), 2 (moderate), or 3 (intense). The expression level of IRS was categorized into negative (0 to 1), mild (2 to 3), moderate (4 to 8), and strongly positive (9 to 12) after calculation. Blood vessel density was defined as the number of vessels in 10 high-power fields (400x).

The expression level was blindly scored by two pathologists (W.-H. H. and C.-C, K.; W.-H.H. is a board-certified veterinary pathologist by the Chinese Society of Veterinary Pathology, and C.-C. K. is a veterinary pathologist trained by the Graduate Institute of Molecular and Comparative Pathobiology). Any discrepancies in the results were evaluated through discussion to determine the immunoreactivity score by observers.

### Clinical outcomes

The clinical outcomes, including recurrence of mast cell tumors, survival status, and cause of death were obtained from the medical records or phone call follow-ups with owners. The follow-up time was at least one year till the end of the study.

### Statistical analysis

The demographic, clinical, and pathological variables were compared between groups. Age, tumor diameter, mitotic count, vessel density, and immune reactivity score were the continuous variables, expressed as the median and interquartile range (IQR). The Shapiro–Wilk test was used to examine the normality for continuous variables. After performing the normality test for each continuous variable, either the Student’s independent t-test or Mann–Whitney U test was utilized to compare the two groups. Breed, sex, number of masses, anatomic location, ulceration, lymph node involvement, WHO clinical stage, histological grade, and YKL-40 expression level were categorical variables. When both variables were categorical, they were compared using a chi-square test or Fisher’s exact test when cells had an expected count of less than 5.

Overall survival (OS) was defined as the time from diagnosis to death from any cause, with censoring applied to dogs alive, lost follow-up, or whose cause of death was other than mast cell tumors. The Kaplan–Meier method was applied to estimate the survival within the study population and between subgroups, with survival comparison conducted using the log-rank test.

The number of cases was based on the high-grade MCT dogs meeting the criteria during the retrospective study period. An equal number of low-grade MCT cases was then selected. The statistical analyses were performed using Prism software, version 10 (GraphPad Software, San Diego, CA, USA). A *p*-value less than 0.05 was considered statistically significant.

## Supplementary Information

Supplementary Material 1.

## Data Availability

The datasets generated and/or analyzed during the current study are not publicly available due to internal regulations but are available from the corresponding author on reasonable request.

## Abbreviations

cMCT: Cutaneous mast cell tumor
HPF: High power field
MC: Mitotic count
MCT: Mast cell tumor
MST: Median survival time
IHC: Immunohistochemistry
IRS: Immunoreactivity score
IQR: Interquartile range
