# Supplementary figures and images for: Prognostic significance of YKL-40 expression in canine cutaneous mast cell tumors
Source: BMC Vet Res. 2024 Nov 29;20:537. doi: 10.1186/s12917-024-04385-1 (PMC11605933; doi:10.1186/s12917-024-04385-1)

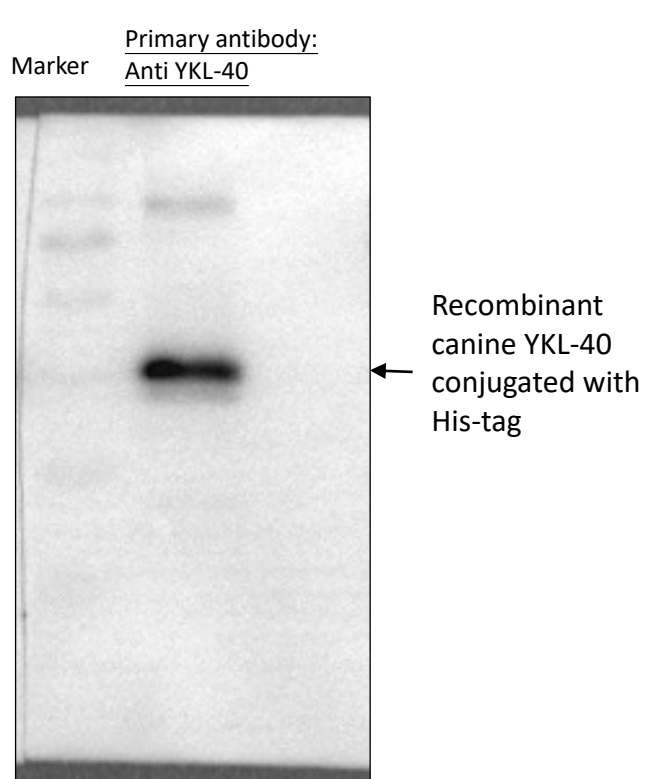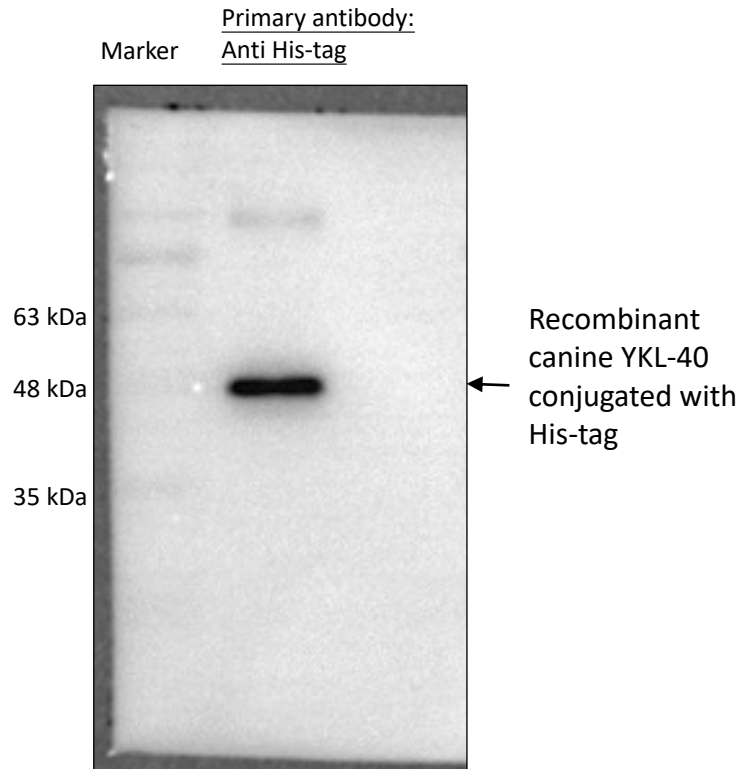

Supplement: Supplementary file 1 — Supplementary Material 1. [file 12917_2024_4385_MOESM1_ESM.pdf]
